# Effect of oral decontamination with chlorhexidine on the incidence of nosocomial pneumonia: a meta-analysis

**DOI:** 10.1186/cc4837

**Published:** 2006-02-20

**Authors:** Lilibeth A Pineda, Ranime G Saliba, Ali A El Solh

**Affiliations:** 1 Division of Pulmonary, Critical Care, and Sleep Medicine, Department of Medicine, University at Buffalo School of Medicine and Biomedical Sciences, Buffalo, NY, USA

## Abstract

**Introduction:**

Nosocomial pneumonia is a significant cause of in-hospital morbidity and mortality. Oral care interventions have great potential to reduce the occurrence of nosocomial pneumonia. Studies using topical antiseptic agents yielded mixed results. We hypothesized that the use of chlorhexidine for oral decontamination would reduce the incidence of nosocomial pneumonia in patients requiring mechanical ventilation.

**Methods:**

This study is a meta-analysis of randomized controlled trials assessing the effect of chlorhexidine on the incidence of nosocomial pneumonia. Data sources were Medline, EMBASE, Cochrane library, citation review of relevant primary and review articles, and contact with expert informants. Out of 1,251 articles screened, 4 randomized, controlled trials were identified that included a total of 1,202 patients. Descriptive and outcome data were extracted by two reviewers independently. Main outcome measures were the incidence of nosocomial pneumonia, and mortality. A random effects model was used.

**Results:**

The incidence of nosocomial pneumonia in the control group was 7% (41 out of 615) compared to 4% (24 out of 587) in the treatment group. Gram-negative bacteria accounted for 78% of the total isolates, with *Pseudomonas aeruginosa *being the most frequently isolated pathogen irrespective of the intervention provided. Duration of mechanical ventilation and intensive care unit length of stay were comparable between the two groups. Overall, the use of oral decontamination with chlorhexidine did not affect the incidence of nosocomial pneumonia (odds ratio of 0.42; 95% confidence interval 0.16–1.06) or the mortality rate (odds ratio 0.77, 95% confidence interval 0.28–2.11).

**Conclusion:**

The use of oral decontamination with chlorhexidine did not result in significant reduction in the incidence of nosocomial pneumonia in patients who received mechanical ventilation, nor altered the mortality rate. The lack of benefit may reflect the few studies conducted in this area. Future trials should focus on a combination strategy of mechanical and pharmacological interventions.

## Introduction

Nosocomial pneumonia (NP) is a frequent complication in critically ill patients requiring mechanical ventilation and is responsible for a significant in-hospital morbidity and mortality. Multiple hospital-associated risk factors for NP have been identified. These risk factors are thought to contribute to increased bacterial colonization of the aerodigestive tract and/or facilitate entry of pathogenic bacteria to the lower respiratory tract. Among these factors are the use of nasogastric tubes, a supine position, re-intubation, manipulation of airway/ventilator circuits, pooling of subglottic secretions, transfusion of packed red blood cells, pH altering agents, and dental plaque colonization [1-4].

While the oral flora of a healthy individual is largely composed of viridans streptococcus, the oral flora undergoes a major shift during intensive care unit (ICU) stay from Gram-positive streptococci to predominantly Gram-negative organisms, including pathogens responsible for NP. The role of these pathogens are highlighted in epidemiological studies showing a high concordance between the bacteria isolated from the oropharyngeal cavity and those recovered from tracheal aspirates [4,5]. Recently, our laboratory, using molecular genotyping, has confirmed this association between pathogens colonizing dental plaques and those responsible for NP in the critically ill institutionalized elderly [6]. As a result, multiple interventional trials have been initiated to assess the efficacy of topical oral antiseptic agents on the incidence of ventilator-associated pneumonia.

Chlorhexidine is an antimicrobial cationic compound active against aerobic and anaerobic bacteria. It increases the bacterial cell wall permeability in a dose dependent fashion by interacting with anionic receptors on the bacterial surface. Its therapeutic benefit has been demonstrated in reducing dental plaque and treating gingivitis [7]. By virtue of its rapid reduction of oropharyngeal bacterial load [8], several studies have evaluated the effectiveness of oral chlorhexidine in preventing NP [9-12]. These trials have yielded conflicting results. Hence, we conducted a meta-analysis of available clinical trials to evaluate the efficacy of oral chlorhexidine application on the incidence of NP in patients who required mechanical ventilation.

## Materials and methods

### Search strategy

We conducted this review in accordance with recommendations put forth by the QUOROM Group [13]. We searched MEDLINE (1966 to August 2005), Biosis Previews (1990 to August 2005), PubMed (mid 1960s to August 2005), EMBASE (January 1990 to August 2005) and Cochrane Library to identify prospective, randomized trials of oral chlorhexidine in patients requiring mechanical ventilation. The following key words were used: chlorhexidine, dental plaques, oropharyngeal decontamination, ventilator, nosocomial, hospital-acquired, health-care acquired pneumonia AND randomized, controlled trials or controlled clinical trials, randomized. In addition, we searched abstracts of conference proceedings, references lists of review articles and retrieved studies. We included studies regardless of date, language, or publication status. The search strategy was conducted iteratively until no new potential randomized controlled trial citations were found on review of the reference lists of retrieved articles.

### Study selection and data extraction

The inclusion criteria were randomized controlled trials in patients requiring mechanical ventilation. We excluded open label, non-comparative, and non-randomized studies. We also excluded studies with the option of combining mechanical and pharmacological oral care for the prevention of dental plaques. Trials that used chlorhexidine as a body or vaginal wash, or endotracheal tubes impregnated with chlorhexidine were excluded. The main outcome measure of the study was the incidence of NP defined as pneumonia occurring 48 hours after hospital admission. Respiratory infections that had no new or progressive radiographic findings were not included. Mortality rate was included as a secondary outcome. Two reviewers screened independently identified titles and abstracts. Potentially relevant studies were retrieved and the full text examined. When important data were not reported, we contacted the authors for information. We assessed reported randomization methods and completeness of data but avoided use of a formal or aggregated score for quality assessment because such use can produce inconsistent results [14]. Discrepancies between reviewers were resolved by consensus.

### Statistical analysis

Incidences of NP and death were treated as dichotomous variables. Data analysis was performed using the random effects model with meta-analysis software (RevMan 4.2; Cochrane collaboration, Oxford, UK). We used risk differences computed on the basis of odds ratios from each of the randomized trials and their respective 95% confidence interval (CI). Statistical heterogeneity for all variables was assessed by using the I^2 ^measure because this measure is independent of the number of studies that are pooled and of the effect-size metric [15]. To assess for possible publication bias, we used the test proposed by Egger and colleagues [16], which provides an assessment of funnel-plot asymmetry (expressed as a *P *value) by applying an inverse-variance weighted approach. For each variable, studies were assigned a Mantel-Haenszel weight that was directly proportional to the sample size and inversely proportional to the variance of each study. A two-sided *P *value less than 0.05 was considered significant.

## Results

Our literature search identified 1,251 potential relevant citations. Of these, we considered seven citations for possible inclusion in the meta-analysis [9-12,17-19]. These seven publications were identified through Medline searches. No unpublished studies, personal communications, or abstract satisfied the inclusion criteria. We excluded two out of the seven studies because they were not randomized and one because it used a single application of chlorhexidine [17-19].

Table 1 provides information on the patients and design of the included studies. Four studies that fulfilled the criteria [9-12]. Two studies [9,11] had a double-blind placebo controlled design and one trial [10] was single-blinded because a comparable placebo to chlorhexidine was not available at the time the study was initiated. The remaining study [12] was a prospective, case-controlled design comparing chlorhexidine to Listerine. Overall, 1,202 patients were enrolled in the selected studies. Two out of the four clinical trials were done on patients undergoing cardiac surgery (coronary aortic bypass surgery or valve replacement surgery) [9,12]. The other two trials enrolled patients from ICUs requiring mechanical ventilation [10,11]. All participants were intubated by oro- or nasotracheal tubes. Patients with tracheostomy were included in one [10] but excluded in the other ICU trial [11]. In the two trials with heart surgeries, antibiotics were administered 12 or 24 hours preoperatively and 48 hours postoperatively as per routine heart surgery protocol [9,12]. Cefuroxime was used in both trials for aortocoronary bypass subjects while vancomycin was provided for those scheduled for valve surgery. In contrast to the trials conducted in ICUs, treatment with chlorhexidine was initiated preoperatively and continued postoperatively. Frequency of chlorhexidine application ranged from twice a day in the ICU group to three times in the group of patients undergoing heart surgery. The duration of treatment varied also from 10 days to 28 days or until extubation, diagnosis of pneumonia, discharge from ICU, or death.

Table 2 shows the clinical characteristics of the patients enrolled in these trials. The mean patient age was 58.5 years. The severity of illness and the dental score index for the critically ill patients were comparable between the controls and the treatment groups. The overall incidence of NP in the chlorhexidine-treated group was 4% (25/587) compared to 7% (41/615) in the control group. Three studies reported on the microbial isolates responsible for the lower respiratory tract infections [9-11]. A total of 20 organisms out of 21 cases were recovered from the treatment groups compared to 30 out of the 39 control cases. Gram-negative bacteria accounted for 78% (39 out of 50) of the total isolates. The distribution of these isolates was comparable among the two groups (46% for the chlorhexidine-treated group and 54% for the control group, *p *= 0.7). The species most commonly represented among the Gram-negatives was *Pseudomonas aeruginosa*. Of the studies that reported on the duration of intubation and length of stay, the weighted mean differences between the treated group and the control group did not reach statistical significance.

**Figure 1:**
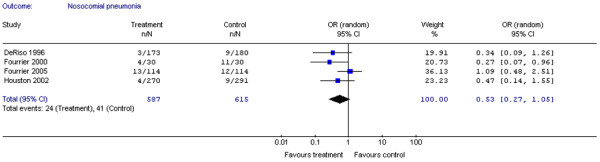
Impact of oral decontamination with chlorhexidine on nosocomial pneumonia. Random effects model. CI, confidence interval; OR, odds ratio.

**Figure 2:**
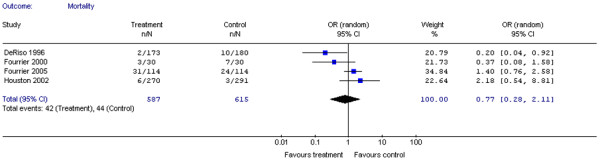
Impact of oral decontamination with chlorhexidine on mortality. Random effects model. CI, confidence interval; OR, odds ratio.

As shown in Figure 1, although the point estimate for the pooled odds ratio favored chlorhexidine treatment in the prevention of NP, this difference was not statistically significant (0.42, 95% CI 0.61–1.06; *p *= 0.07). Similarly, there were also no significant difference in mortality rate between the two groups (0.77, 95% CI 0.28–2.11; *p *= 0.6; Figure 2). For these estimates, we found no evidence of statistical heterogeneity or publication bias (*p *= 0.13 and *p *= 0.68 for the incidence of pneumonia and mortality, respectively).

## Discussion

The results of this meta-analysis suggest that the incidence of NP and rate of mortality are not reduced by administration of the oral antiseptic agent chlorhexidine. Of the four trials that were selected for inclusion in the meta-analysis, only one trial showed a statistically significant reduction in the incidence of NP [10]; yet, the study failed to adjust for inclusion of repeated observations. Moreover, DeRiso and coworkers [9] reported a 69% reduction in overall nosocomial respiratory infections, but when a comparison of the rate of lower respiratory tract infections was presented, the difference between the treatment and control groups was not statistically significant. If chlorhexidine treatment has been proven to be an effective therapy *in vitro *for eradication of bacteria responsible for oropharyngeal colonization, why did it not improve the rate of lower respiratory tract infections in mechanically ventilated patients?

A review of studies that have examined oral and lung colonization have shown that changes in the microenvironment of the oral cavity likely play a key role in the colonization of the oropharynx with NP related pathogens [20,21]. Serial examination of dental plaques of critically ill patients revealed that while the frequency of dental colonization increased in critically ill patients, the density of bacterial pathogens following treatment with chlorhexidine remained stable [11]. The lack of a complete decontaminating effect of chlorhexidine on dental plaques might suggest, among other things, impairment of innate oral immunity and/or loss of protective function of saliva. Because salivary flow provides a mechanical tool for removal of plaques and microorganisms, reduced circulation of saliva leads to microbial overgrowth, accumulation of dental plaques, and rampant dental caries [22,23]. This constant buildup of dental plaques might explain the failure to completely eradicate microorganisms with chlorhexidine treatment.

Formation of biofilm is another potential microenvironmental factor that may influence the eradication of ventilator associated pneumonia related pathogens from dental plaques. Fourrier and colleagues [11] noted that microbiological analysis of dental plaques obtained from patients with late onset ventilator associated pneumonia revealed a high prevalence of highly resistant bacterial pathogens (*Pseudomonas*, *Acinetobacter*, and *Enterobacter *species) that were not eliminated by topical chlorhexidine. Notoriously, upon attachment, *P. aeruginosa *activates a set of genes responsible for the release of diffusible homoserine lactones (quorum sensors). These organic molecules promote biofilm formation, which protects bacteria from host defenses and antibiotics [24] and prevents antiseptic agents from reaching the bacteria embedded in the dental plaques.

If reducing bacterial growth in the dental plaques with chlorhexidine did not result in a significant reduction in NP, perhaps there are other unrecognized niches for respiratory pathogens between the oropharynx and the lungs that are implicated in the development of lower respiratory tract infections. It has been shown that the lungs of normal animals are not able to clear bacteria present in the form of biofilm fragments enclosed in artificial matrices [25]. As such, endotracheal tubes may serve as foci for bacteria in the biofilm that invariably forms on the inner lumen. Once the bacteria are well established, the bacterial burden attains high levels, and the biofilm, when fractured and displaced into the lower airways, acts as inoculum for the development of pneumonia [26]. Unless they are able to eradicate such biofilms, oral antiseptic agents alone might fall short of attaining their objective.

Our analysis has several important limitations. First, there were major differences between the studies conducted in the cardiac surgery population [9,12] versus those performed in ICU settings [10,11]. Patients admitted for elective cardiac surgery were likely to have different comorbid conditions and better physiological status at the time of intubation than were patients intubated emergently. Moreover, the length of mechanical ventilation for cardiac surgery and ICU patients would be significantly different such that colonization with highly resistant bacteria in ICU patients would be less amenable to chlorhexidine treatment [11]. Second, all of the trials for the meta-analysis were conducted in academic teaching centers, and so it is unclear if these results are generalizable to other institutions. Third the trials used different approaches for the control arms. Two investigations [9,11] used placebos that were completely indistinguishable from chlorhexidine by color, taste, and odor, whereas the other trials relied on either standard oral care or Listerine. This may have resulted in confounding when the data were pooled. Fourth, even though we were able to pool results across four trials, the combined sample size may still have been inadequate for detecting important clinical differences.

## Conclusion

In this meta-analysis, we failed to find any clinical benefits of regular oral chlorhexidine application on the incidence of NP and mortality rate in critically ill patients requiring mechanical ventilation. Although colonization of dental plaques with pathogenic bacteria may be a precursor for the disease, chlorhexidine based decontamination of oral microbial flora alone might not be sufficient to reduce the burden of bacteria responsible for NP. Routine oral care in ICU settings should be pursued along with other preventive measures aimed at reducing biofilm formation pending the results of ongoing trials addressing oral mechanical interventions and silver coated endotracheal tubes.

## Key messages

• 	Dental plaques are considered an important reservoir for pathogenic bacteria associated with lower respiratory tract infection.

• 	Topical administration of bactericidal agents is effective in controlling dental plaque formation in critically ill patients.

• 	The use of topical chlorhexidine does not result in significant reduction in the incidence of NP in mechanically ventilated patients.

• 	Additional research is needed to determine the effectiveness of combined chemical and mechanical interventions on the rate of NP.

## Abbreviations

CI = confidence interval; ICU = intensive care unit; NP = nosocomial pneumonia.

## Competing interests

The authors declare that they have no competing interests.

## Authors' contributions

LP conducted the literature search, performed the statistical analysis, and drafted the manuscript. RS assisted in the literature search and entered the data into the designated software. AES conceived of the study, reviewed all selected studies, and edited the manuscript. All authors read and approved the final manuscript.

## Figures and Tables

**Table 1:** Characteristics of the randomized trials included in the meta-analysis

Reference	Year	Type	Number of patients	Intervention
[9]	1996	Heart surgery	353	0.12% CHX oral rinse BID
[10]	2000	ICU	60	0.2% CHX gel TID
[12]	2002	Heart surgery	561	0.12% CHX oral rinse BID
[11]	2005	ICU	228	0.2% CHX gel TID

BID, twice a day; CHX, chlorhexidine; ICU, intensive care unit; TID, three times a day.

**Table 2:** Clinical characteristics and outcome measures of trials included in the meta-analysis

Reference	SAPS II		CAO dental index		Incidence of pneumonia		ICU LOS (days)		Mortality rate	
	T	C	T	C	T	C	T	C	T	C

[9]	NR	NR	NR	NR	3/173	9/180	7.9	8.5	2/173	10/180
[10]	37 ± 15	33 ± 13	16 ± 7	18 ± 8	4/30	11/30	18 ± 16	24 ± 19	3/30	7/30
[12]	NR	NR	NR	NR	4/270	9/291	14 ± 9	13 ± 9	6/270	3/291
[11]	45 ± 18	45 ± 18	19 ± 9	20 ± 9	13/114	12/114	NR	NR	31/114	24/114

C, control group; CAO, caries-absent-occluded; ICU, intensive care unit; LOS, length of stay; NR, not reported; SAPS, Simplified Acute Physiology Score; T, treatment group.

## References

[B1] Kollef MH (2004). Prevention of hospital-associated pneumonia and ventilator-associated pneumonia. Crit Care Med.

[B2] Osmon SB, Kollef MH (2005). Prevention of pneumonia in the hospital setting. Clin Chest Med.

[B3] Arozullah A, Khuri S, Henderson W, Daley J, Participants in the National Veterans Affairs Surgical Quality Improvement Program (2001). Development and validation of a multi-factorial risk index for predicting postoperative pneumonia after major noncardiac surgery. Ann Intern Med.

[B4] Fourrier F, Duvivier B, Boutigny H, Rourrel-Delvallez M, Chopin C (1998). Colonization of dental plaque: a source of nosocomial infections in intensive care unit patients. Crit Care Med.

[B5] Garrouste-Ortegas M, Chevret S, Arlet G, Marie O, Rouveau M, Popoff N, Schlemmer B (1997). Oropharyngeal or gastric colonization and nosocomial pneumonia in adult intensive care unit patients: a prospective study based on genomic DNA analysis. Am J Respir Crit Care Med.

[B6] El-Solh A, Pietrantoni C, Bhat A, Okada M, Zambon J, Aquilina A, Berbary E (2004). Colonization of dental plaques: A reservoir of respiratory pathogens for hospital-acquired pneumonia in institutionalized elders. Chest.

[B7] Munro C, Grap MJ (2004). Oral health and care in the intensive care unit: state of the science. Am J Crit Care.

[B8] Veksler A, Kayrouz G, Newman M (1991). Reduction of salivary bacteria by preprocedural rinses with chlorhexidine 0.12%. J Periodontol.

[B9] DeRiso A, Ladowski J, Dillon T, Justice J, Peterson A (1996). Chlorhexidine gluconate 0.12% oral rinse reduces the incidence of total nosocomial respiratory infection and nonprophylactic systemic antibiotic use in patients undergoing heart surgery. Chest.

[B10] Fourrier F, Cau-Pottier E, Boutigny H, Roussel-Delvallez M, Jourdain M, Chopin C (2000). Effects of dental plaque antiseptic decontamination on bacterial colonization and nosocomial infections in critically ill patients. Intensive Care Med.

[B11] Fourrier F, Dubois D, Pronnier P, Herbecq P, Leroy O, Desmettre T, Pottier-Cau E, Boutigny H, Di Pompeo C, Durocher A, Roussel-Delvallez M, for the PIRAD Study Group (2005). Effect of gingival and dental plaque antiseptic decontamination on nosocomial infections acquired in the intensive care unit: A double-blind placebo controlled multicenter study. Crit Care Med.

[B12] Houston S, Hougland P, Anderson J, LaRocco M, Kennedy V, Gentry L (2002). Effectiveness of 0.12% chlorhexidine gluconate oral rinse in reducing prevalence of nosocomial pneumonia in patients undergoing heart surgery. Am J Crit Care.

[B13] Moher D, Cook DJ, Eastwood S, Olkin I, Rennie D, Stroup DF (1999). Improving the quality of reports of meta-analyses of randomized controlled trials: the QUOROM statement. Quality of Reporting of Meta-analyses. Lancet.

[B14] Juni P, Witschi A, Bloch R, Egger M (1999). The hazards of scoring the quality of clinical trials for meta-analysis. JAMA.

[B15] Higgins JP, Thompson SG (2002). Quantifying heterogeneity in a meta-analysis. Stat Med.

[B16] Egger M, Davey Smith G, Schneider M, Minder C (1997). Bias in meta-analysis detected by a simple, graphical test. BMJ.

[B17] Genuit T, Bochicchio G, Napolitano L, McCarter R, Roghman M (2001). Prophylactic chlorhexidine oral rinse decreases ventilator-associated pneumonia in surgical ICU patients. Surg Infect (Larchmt).

[B18] Rodriguez Artalejo F, Garcia Caballero J, Aguado Matorras A, del Rey Calero R (1987). Oral lavage with chlorhexidine and hospital pneumonia. Med Clin (Barc).

[B19] Grap M, Munro C, Elswick R, Sessler C, Ward K (2004). Duration of action of a single early oral application of chlorhexidine on oral microbial flora in mechanically ventilated patients: A pilot study. Heart Lung.

[B20] Bonten MJ, Bergmans DC, Ambergen AW, de Leeuw PW, van der Geest S, Stobberingh EE, Gaillard CA (1996). Risk factors for pneumonia and colonization of respiratory tract and stomach in mechanically ventilated ICU patients. Am J Respir Crit Care Med.

[B21] Sole ML, Poalillo FE, Byers JF, Ludy JE (2002). Bacterial growth in secretions and on suctioning equipment of orally intubated patients: a pilot study. Am J Crit Care.

[B22] Longman L, Higham S, Bucknall R, Kaye S, Edgar W, Field E (1997). Signs and symptoms in patients with salivary gland hypofunction. Postgrad Med J.

[B23] Loesche W, Schork A, Terpenning M, Chen Y, Stoll J (1995). Factors which influence levels of selected organisms in saliva of older individuals. J Clin Microbiol.

[B24] Prince AS (2002). Biofilms, antimicrobial resistance, and airway infection. N Engl J Med.

[B25] Costerton W, Veeh R, Shirtliff M, Pasmore M, Post C, Ehrlich G (2003). The application of biofilm science to the study and control of chronic bacterial infections. J Clin Invest.

[B26] Adair C, Gorman S, Feron B, Byers LM, Jones DS, Goldsmith CE, Moore JE, Kerr JR, Curran MD, Hogg G (1999). Implications of endotracheal tube biofilm for ventilator-associated pneumonia. Intensive Care Med.

